# Ki-67 labeling index predicts tumor progression patterns and survival in patients with atypical meningiomas following stereotactic radiosurgery

**DOI:** 10.1007/s11060-023-04537-7

**Published:** 2024-02-18

**Authors:** Motoyuki Umekawa, Yuki Shinya, Hirotaka Hasegawa, Ramin A. Morshed, Atsuto Katano, Aya Shinozaki-Ushiku, Nobuhito Saito

**Affiliations:** 1 Department of Neurosurgery, The University of Tokyo Hospital, Tokyo, 113-8655 Japan; 2 Department of Neurologic Surgery, Mayo Clinic, Rochester, MN USA; 3 Department of Radiology, The University of Tokyo Hospital, Tokyo, Japan; 4 Department of Pathology, The University of Tokyo Hospital, Tokyo, Japan

**Keywords:** Atypical meningioma, Ki-67 labeling index, Recurrence pattern, Stereotactic radiosurgery

## Abstract

**Purpose:**

This study investigated whether Ki-67 labeling index (LI) correlated with clinical outcomes after SRS for atypical meningiomas.

**Methods:**

This retrospective study examined 39 patients with atypical meningiomas who underwent SRS over a 10-year study period. Ki-67 LI was categorized into 3 groups: low (< 5%), intermediate (5%–10%), and high (> 10%). Local tumor control rates (LCRs), progression-free rates (PFRs), disease-specific survival (DSS) rates, and adverse radiation-induced events (AREs) were evaluated.

**Results:**

The median follow-up periods were 26 months. SRS was performed at a median prescription dose of 18 Gy for tumors with a median Ki-67 LI of 9.6%. The 3-year LCRs were 100%, 74%, and 25% in the low, intermediate, and high LI groups, respectively (*p* = 0.011). The 3-year PFRs were 100%, 40%, and 0% in the low, intermediate, and high LI groups (*p* = 0.003). The 5-year DSS rates were 100%, 89%, and 50% in the low, intermediate, and high LI groups (*p* = 0.019). Multivariable Cox proportional hazard analysis showed a significant correlation of high LI with lower LCR (hazard ratio [HR], 3.92; 95% confidence interval [CI] 1.18–13.04, *p* = 0.026), lower PFR (HR 3.80; 95% CI 1.46–9.88, *p* = 0.006), and shorter DSS (HR 6.55; 95% CI 1.19–35.95, *p* = 0.031) compared with intermediate LI. The ARE rates were minimal (8%) in the entire group.

**Conclusion:**

Patients with high Ki-67 LI showed significantly more tumor progression and tumor-related death. Ki-67 LI might offer valuable predictive insights for the post-SRS management of atypical meningiomas.

**Supplementary Information:**

The online version contains supplementary material available at 10.1007/s11060-023-04537-7.

## Introduction

Atypical meningiomas account for approximately 15% of all meningiomas and are diagnosed with a pathological confirmation of 4–19 high mitotic cells per 10 high power fields or brain invasion, categorized in the 2021 World Health Organization (WHO) Classification of Tumor of Central Nervous System as grade 2 [1, 2]. Diagnosis is confirmed based on the number of mitoses observed as well as brain invasion on histological examination. Overall, the prognosis is worse than that of WHO grade 1 meningiomas with higher recurrence rates seen, varying between 38 to 66% in previous studies [3–6]. Fractionated radiotherapy for postoperative remnants is recommended based on guidelines from the European Association of Neuro-Oncology [1, 7]. However, postoperative management of atypical meningiomas remains controversial, and there is debate whether to observe patients after gross total resection or to perform upfront adjuvant radiotherapy to the resection site. Additionally, there is a lack of consensus regarding the optimal management of tumor recurrences [8–11]. In light of this, a more detailed classification based on post-treatment outcomes is required.

Stereotactic radiosurgery (SRS) is an effective treatment option for meningiomas because of its ability to deliver highly conformal and focused doses to the tumor margin while sparing surrounding neurovascular structures [12]. Several studies have already demonstrated the effectiveness of upfront adjuvant SRS for atypical meningiomas [13–25]. Those results indicated that tumor recurrence could be classified as intrafield recurrence (tumor progression within the irradiated area) and marginal or remote recurrence (tumor progression outside the irradiation area) [25, 26]. Moreover, multiple radiotherapy treatments for frequent recurrences can increase the risks of adverse radiation effects (AREs) [13, 15]. Hence, it may be beneficial to predict not just the response to SRS but also the pattern of recurrence in atypical meningiomas to determine if SRS treatment paradigms need to be modified.

The Ki-67 labeling index (LI) is a valuable tool that reflects tumor proliferation capability and has been shown to correlate with progression after surgery for benign and high-grade meningiomas [27–31]. Recent studies have reported that it can correlate with progression of benign and high-grade meningiomas after SRS [13, 32]. Increased Ki-67 LI found in atypical meningiomas might serve as a predictor of recurrence [33]. However, the use of Ki-67 LI to assess the outcomes following SRS in atypical meningiomas is unclear. This study aimed to evaluate outcomes after SRS for atypical meningiomas and stratify the risk of tumor progression, recurrence patterns, and disease-specific survival (DSS) based on Ki-67 LI.

## Methods

### Patients and tumor characteristics

The clinical data of 451 consecutive patients with meningiomas treated with single-session SRS between June 1992 and February 2022 at our institution were collected from an institutional Gamma Knife database. Among these patients, 47 patients with atypical meningiomas were identified, and patients with a follow-up period of < 3 months (n = 8) were excluded. In total, 39 atypical meningiomas were analyzed for this study. Patients who underwent external-beam radiotherapy before SRS were included. Tumor tissue from all surgically obtained specimens were reviewed, and all diagnoses were confirmed based on the 2016 WHO Classification of Tumors of the Central Nervous System criteria. Tissue from seven tumors that were resected and diagnosed as atypical meningioma before 2007 were re-evaluated and reconfirmed to meet the 2016 criteria [2, 34]. Formalin-fixed, paraffin-embedded specimens were subjected to immunohistochemistry for Ki-67, and the LI within the hotspot was calculated under a light microscope by board-certified pathologists. All participants in the present study provided written informed consent, and all components of this study were authorized by the appropriate Institutional Review Board (The Research Ethics Committee of our institution, #2231).

### SRS procedures and techniques

SRS was performed using a Leksell GammaKnife (Elekta AB, Stockholm, Sweden). After head fixation using a Leksell frame (Elekta Instruments, Stockholm, Sweden), stereotactic imaging (computed tomography [CT] before July 1996, magnetic resonance imaging [MRI] between August 1996 and January 2018, and cone-beam CT thereafter) was performed to obtain the precise three-dimensional coordinates. Thin-slice MR images (gadolinium-contrasted T1-weighted images and fast imaging employing steady-state acquisition) obtained the day before SRS were co-registered to define the tumor, surrounding cranial nerves, and vasculature. Radiosurgical treatment was planned and approved by the dedicated neurosurgeons and radiation oncologists involved with the procedure. All treatment planning was performed using commercially available software programs (KULA treatment planning system until 1998 and Leksell GammaPlan® [Elekta Instruments]).

### Follow-up and treatment outcomes

**Fig. 1:**
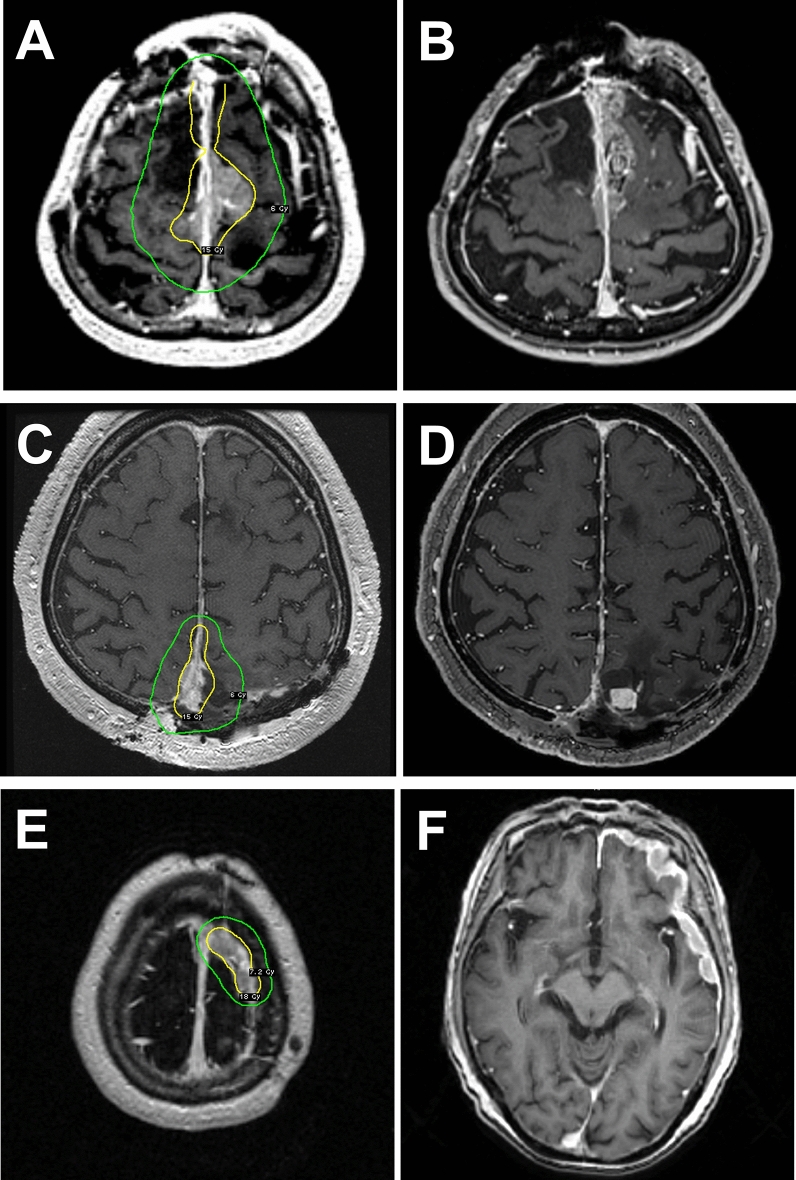
Demonstrative cases representing intrafield recurrence, marginal recurrence, and remote recurrence. **A**, **B** “Intrafield recurrence” was defined as a > 10% increase in volume inside the 50% isodose line. **C**, **D** “Marginal recurrence” was defined as new tumor progression between 50 and 20% isodose lines. **E**, **F** “Remote recurrence,” defined as new tumor progression outside the 20% isodose line, was also collected. Yellow lines indicate 50% isodose, and green lines indicate 20% isodose

**Fig. 2:**
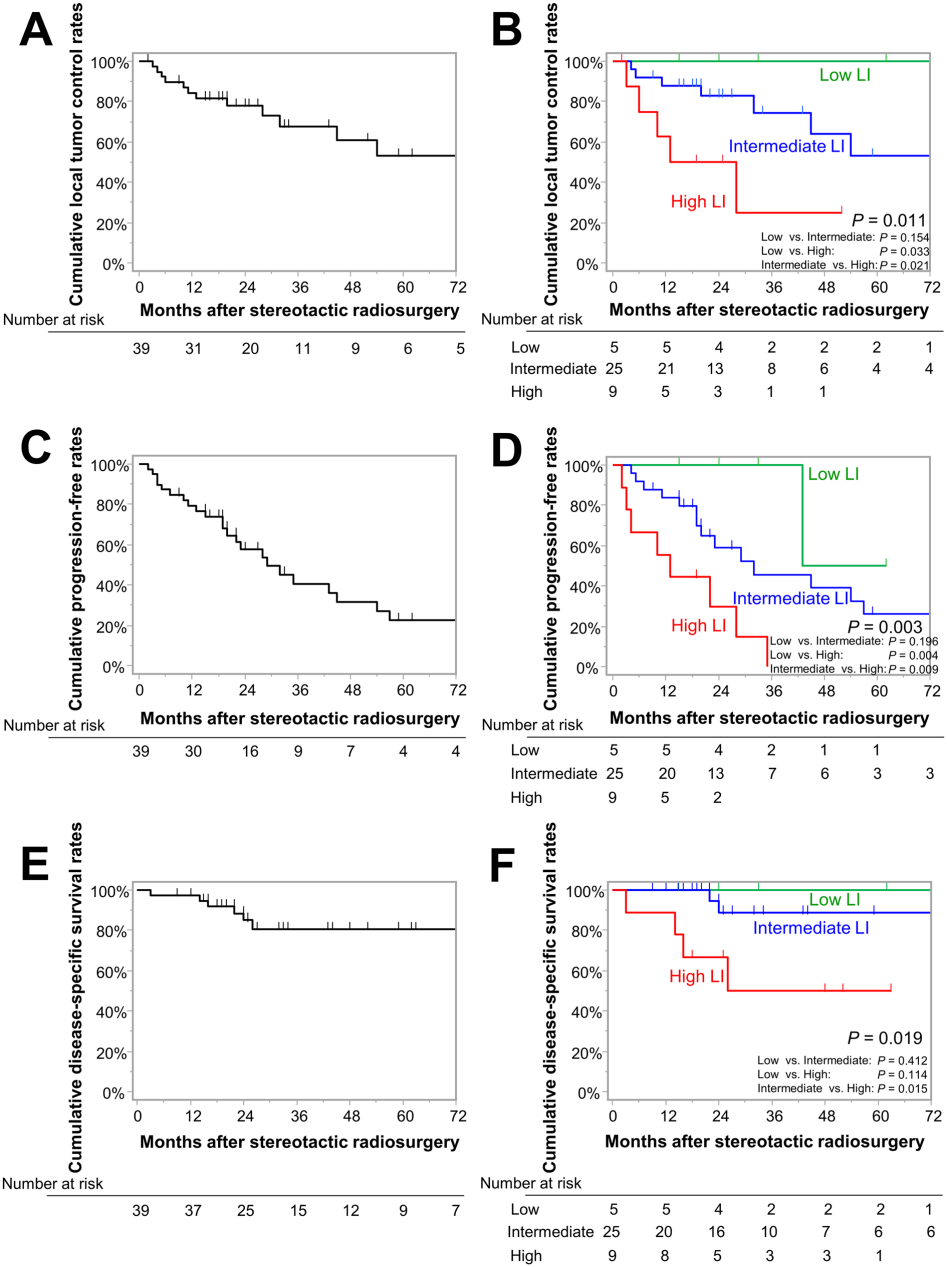
Kaplan–Meier curves for: **A**, **B** local tumor control rates for the entire cohort and compared between three groups stratified by a Ki-67 labeling index of < 5%, 5%–10%, and > 10%, **C**, **D** progression-free rates for the entire cohort and compared between three groups stratified by a Ki-67 labeling index of < 5%, 5%–10%, and > 10%, **E**,** F** disease-specific survival rates for the entire cohort and compared between three groups stratified with a Ki-67 labeling index of < 5%, 5%–10%, and > 10%

Clinical evaluations and MRI were performed every 3–6 months after the date of SRS. Data on tumor status and SRS-related complications were prospectively collected for the institutional Gamma Knife database. Two neurosurgeons and two neuroradiologists independently assessed radiologic evidence of recurrence. Tumor progression was defined as one of three types of events: (1) “intrafield recurrence,” defined as a > 10% increase in volume inside the 50% isodose line on two or more consecutive imaging studies (Fig. 1A, B), (2) “marginal recurrence,” defined as new tumor progression between the lines of 50% and 20% isodose (Fig. 1C, D), and (3) “remote recurrence,” defined as new tumor progression outside the 20% isodose line (Fig. 1E, F) [25, 26, 32]. AREs were graded according to the Common Terminology Criteria for Adverse Events (CTCAE) version 5.0. Peritumoral T2 signal change expanding beyond a 2-mm margin from the tumor after SRS was also considered to be an ARE.

### Statistical analyses

Baseline patient, tumor, and SRS dosimetry characteristics were summarized. Continuous variables were presented as medians and interquartile ranges (IQRs), while categorical variables were presented as numbers and percentages. The Kaplan–Meier method was used to evaluate therapeutic effects, with outcomes comprising local tumor control rates (LCRs), progression-free rates (PFRs), and DSS rates. Local tumor control was defined as the absence of intrafield recurrence, and progression-free status was defined as the absence of any recurrence pattern including a lack of intrafield, margin, or remote recurrence. DSS was defined as the absence of any mortality associated with the treated tumors. To further analyze the association between Ki-67 LI and SRS outcomes, the patients were classified into three groups based on Ki-67 LI: low LI (< 5%), intermediate LI (5%–10%), and high LI (> 10%) groups, based on the previous studies on Ki-67 LI in meningiomas [33, 35]. The LCRs, PFRs, and DSS rates between groups were compared using the log-rank test, and multiple curves were compared using the log-rank test with the Bonferroni correction. In addition, receiver operating curve (ROC) analyses on Ki-67 LI were performed to calculate each area under the curve (AUC), sensitivity, and specificity on intrafield recurrence, any progression, and disease-specific mortality, and to indicate a cut-off Ki-67 LI increasing each risks using Youden index. Continuous variables (age, maximum diameter, target volume, and radiosurgical dose) were entered into the models after dichotomization using the median values. Factors associated with local tumor recurrence and AREs were examined using bivariate and multivariable Cox proportional hazards analyses. Statistical significance was set at *p* < 0.05. All statistical analyses were performed using the JMP® Pro 17.0.0 software (SAS Institute Inc., Cary, NC, USA).

## Results

### Baseline characteristics

Baseline patient characteristics and dosimetry data are summarized in Table 1. The median (IQR) age and follow-up period were 73 (63–77) years and 26 (18–59) months, respectively. The minimum follow-up period among alive patients at the last visit was 9 months. The median (IQR) Ki-67 LI was 9.6% (5.0–10.0%), with 5, 25, and 9 tumors in the low (< 5%), intermediate (5%–10%), and high (> 10%) LI groups, respectively. Postoperative adjuvant SRS was performed for seven (18%) patients after tumor resection before any noted progression. External-beam radiotherapy was performed for 13 (33%) patients before SRS. The median (IQR) central and prescription doses were 36 Gy (32–40 Gy) and 18 Gy (16–18 Gy), respectively.

**Table 1:** Baseline characteristics and dosimetry data for the entire cohort and stratified by the Ki-67 labeling index (LI) groups

Variables	All (n = 39)	Low LI group (n = 5)	Intermediate LI group (n = 25)	High LI group (n = 9)	*p-*value
Median [interquartile range], N (%)					
Ki-67 LI, %	9.6 [5.0–10.0]	4.0 [3.0–4.0]	8.0 [5.0–10.0]	20.0 [15.0–27.5]	0.001^*^
Age at SRS, years	73 [63–77]	75 [62–81]	74 [67–78]	67 [61–72]	0.244
Female sex	19 (49%)	2 (40%)	13 (52%)	4 (44%)	0.850
Follow-up period, months	26 [18–59]	33 [20–72]	27 [20–66]	25 [15–50]	0.574
History of radiation therapy	13 (33%)	2 (40%)	7 (28%)	4 (44%)	0.631
Upfront adjuvant	11 (24%)	0 (0%)	4 (16%)	3 (33%)	0.195
Maximum tumor diameter, mm	33 [22–45]	45 [23–53]	31 [21–41]	33 [26–51]	0.477
Target volume, mL	7.5 [3.8–15.4]	10.3 [5.6–16.8]	7.2 [3.7–12.4]	7.5 [4.1–22.1]	0.463
Central dose, Gy	36 [32–40]	36 [33–38]	36 [32–40]	36 [34–43]	0.443
Prescription dose, Gy	18 [16–18]	18 [14–19]	16 [16–18]	18 [17, 18]	0.563
Locations					0.062
Convexity	7 (18%)	0 (0%)	3 (12%)	4 (44%)	
Parasagittal/falx	11 (28%)	0 (0%)	9 (36%)	2 (22%)	
Anterior skull base	4 (12%)	2 (40%)	2 (8%)	0 (0%)	
Middle skull base	12 (31%)	3 (60%)	7 (28%)	2 (22%)	
Posterior skull base	5 (13%)	0 (0%)	4 (16%)	1 (11%)	

*LI* labeling index, *N* number, *SRS* stereotactic radiosurgery

*p-values of < 0.05 are considered significant

### Tumor control

In the entire cohort, 14 (36%) patients experienced intrafield recurrence, and 12 (31%) experienced marginal recurrence (**Supplementary Table 1**). One patient experienced intrafield and marginal recurrence simultaneously. Remote recurrence was observed in three (8%) patients, and all lesions were detected simultaneously with marginal recurrence. In the entire cohort, the cumulative 1-, 3-, and 5-year LCRs were 84%, 68%, and 53%, respectively (Fig. 2A); and the PFRs were 79%, 41%, and 23%, respectively (Fig. 2C).

Using the three groups stratified by Ki-67 LI, the 1-, 3-, and 5-year LCRs with low LI were all 100%, and LCRs with intermediate LI were 88%, 74%, and 53%, respectively; the 1- and 3-year LCRs with high LI were 63% and 25%, respectively (Fig. 2B; *p* = 0.011). Differences between low and high LI (*p* = 0.033) and between intermediate and high LI (*p* = 0.021) were marginally significant. The ROC analysis for intrafield recurrence and Ki-67 LI showed an AUC of 0.797 (Supplementary Fig. 1). The Youden index identified a cutoff at Ki-67 LI = 8%, with a sensitivity of 1.000 and specificity of 0.600. Bivariate and multivariable regression analyses were performed to determine if Ki-67 LI was associated with recurrence. In addition to Ki-67 LI, sex and tumor volume were used for multivariable analysis. Tumors with high Ki-67 LI were associated with greater intrafield recurrence risk using bivariate (hazard ratio [HR] 3.75, 95% confidence interval [CI] 1.13–12.49, *p* = 0.031; Table 2) and multivariable (HR 6.10, 95% CI 1.56–23.95, *p* = 0.010; Table 2) analyses. Larger tumor volume was associated with greater intrafield recurrence risk only in multivariable analysis (HR 1.08, 95% CI 1.01–1.16, *p* = 0.023; Table 2). Furthermore, 1-, 3-, and 5-year PFRs with low, and intermediate LI were 100%, 100%, and 50%; and 84%, 46%, and 26%, respectively; the 1- and 3-year PFRs with high LI were 56% and 0%, respectively (Fig. 2D *p* = 0.003). Differences between low and high LI (*p* = 0.009) and between intermediate and high LI (*p* = 0.004) were significant as well. The ROC analysis for any progression and Ki-67 LI showed an AUC of 0.793 (Supplementary Fig. 1). The Youden index indicated a cutoff at Ki-67 LI = 7%, with sensitivity of 0.880 and specificity of 0.714. Tumors with high Ki-67 LI were identified as risk factors of lower PFRs using bivariate (high vs. low, HR 11.97, 95% CI 1.46–98.04, *p* = 0.021: high vs. intermediate, HR 3.43, 95% CI 1.35–8.70, *p* = 0.010; Table 3) and multivariable (high vs. low, HR 12.71, 95% CI 1.51–107.23, *p* = 0.019: high vs. intermediate, HR 4.12, 95% CI 1.54–11.04, *p* = 0.005; Table 3) analyses in which tumor volume and indication of SRS in addition to Ki-67 LI were included. Tumor volume was not associated with lower PFRs.

**Table 2:** Results of bivariate and multivariate analyses for local tumor progression after stereotactic radiosurgery

	Bivariate		Multivariate	
	HR [95% CI]	*p-*value	HR [95% CI]	*p-*value
Age, years (continuous)	1.02 [0.97–1.08]	0.422	–	–
Age > 70 years (vs. ≤ 70 years)	1.00 [0.33–3.02]	0.994	–	–
Male (vs. female)	0.49 [0.15–1.64]	0.249	0.37 [0.11–1.31]	0.123
Convexity, midline (vs. skull base)	0.77 [0.26–2.29]	0.634	–	–
Maximum diameter, mm (continuous)	1.02 [0.98–1.08]	0.270	–	–
Maximum diameter > 35 mm (vs. ≤ 35 mm)	1.44 [0.48–4.33]	0.521	–	–
Volume, mL (continuous)	1.06 [0.99–1.13]	0.095	1.08 [1.01–1.16]	0.023^*^
Volume > 8 mL (vs. ≤ 8 mL)	0.95 [0.32–2.86]	0.934	–	–
Ki-67 LI, % (continuous)	1.04 [0.97–1.10]	0.196	–	–
High LI (vs. low LI)	NA	NA	NA	NA
High LI (vs. intermediate LI)	3.75 [1.13–12.49]	0.031^*^	6.10 [1.56–23.95]	0.010^*^
History of radiation therapy	1.34 [0.43–4.13]	0.611	–	–
Salvage SRS (vs. adjuvant SRS)	0.61 [0.17–2.24]	0.460	–	–
Central dose, Gy (continuous)	1.0 [0.90–1.13]	0.932	–	–
Central dose > 36 Gy (vs. ≤ 36 Gy)	0.99[0.32–3.05]	0.984	–	–
Marginal dose, Gy (continuous)	0.95 [0.72–1.25]	0.736	–	–
Marginal dose > 18 Gy (vs. ≤ 18 Gy)	1.00 [0.33–3.03]	0.998	–	–

*CI* confidence interval, *HR* hazard ratio, *LI* labeling index, *NA* not adequately calculated, *SRS* stereotactic radiosurgery

*p-values of < 0.05 are considered significant;

**Table 3:** Results of bivariate and multivariate analyses for tumor progression after stereotactic radiosurgery

	Bivariate		Multivariate	
	HR [95% CI]	*p-*value	HR [95% CI]	*p-*value
Age, years (continuous)	1.02 [0.98–1.06]	0.385	–	–
Age > 70 years (vs. ≤ 70 years)	1.03 [0.46–2.32]	0.942	–	–
Male (vs. female)	1.23 [0.53–2.83]	0.628	–	–
Convexity, midline (vs. skull base)	0.88 [0.39–1.96]	0.748	–	–
Maximum diameter, mm (continuous)	1.00 [0.97–1.03]	0.858	–	–
Maximum diameter > 35 mm (vs. ≤ 35 mm)	0.78 [0.33–1.73]	0.511	–	–
Volume, mL (continuous)	1.02 [0.97–1.08]	0.378	1.03 [0.97–1.09]	0.315
Volume > 8 mL (vs. ≤ 8 mL)	0.85 [0.38–1.92]	0.694	–	–
Ki-67 LI, % (continuous)	1.04 [1.00–1.09]	0.047^*^	–	–
High LI (vs. low LI)	11.97 [1.46–98.04]	0.021^*^	12.71 [1.51–107.23]	0.019^*^
High LI (vs. intermediate LI)	3.43 [1.35–8.70]	0.010^*^	4.12 [1.54–11.04]	0.005^*^
History of radiation therapy	0.69 [0.27–1.75]	0.438	–	–
Salvage SRS (vs. adjuvant SRS)	0.50 [0.20–1.29]	0.152	0.59 [0.21–1.64]	0.309
Central dose, Gy (continuous)	1.04 [0.96–1.13]	0.395	–	–
Central dose > 36 Gy (vs. ≤ 36 Gy)	1.42 [0.63–3.19]	0.401	–	–
Marginal dose, Gy (continuous)	1.15 [0.94–1.40]	0.160	–	–
Marginal dose > 18 Gy (vs. ≤ 18 Gy)	0.54 [0.12–2.33]	0.408	–	–

*CI* confidence interval, *HR* hazard ratio, *LI* labeling index, *SRS* stereotactic radiosurgery

*p-values of < 0.05 are considered significant

### DSS

Among the 39 patients, 29 (74%) were alive by last follow-up, 7 (18%) had died of uncontrolled tumor progression, and 3 (8%) had died from unrelated causes. The cumulative 1-, 3-, and 5-year DSS rates were 97%, 81%, and 81%, respectively (Fig. 2E). Stratified by Ki-67 LI, the 1-, 3-, 5-year DSS rates with low LI were all 100%, those with intermediate LI were 100%, 100%, and 89%, respectively; and those with high LI were 89%, 50%, and 50%, respectively (*p* = 0.019; Fig. 2F). The ROC analysis for disease-specific mortality and Ki-67 LI showed an AUC of 0.766 (Supplementary Fig. 1). The Youden index identified a cutoff at Ki-67 LI = 8%, with sensitivity of 1.000 and specificity of 0.469. A significant difference in DSS rates between intermediate and high LI groups was identified (*p* = 0.015), and high LI compared with intermediate LI was identified as a risk factor related to shorter DSS using bivariate (HR 6.27, 95% CI 1.14–34.26, *p* = 0.034; Supplementary Table** 2**) and multivariable (HR 6.55, 95% CI 1.19–35.95, *p* = 0.035; Supplementary Table 2) analyses in which tumor volume and Ki-67 LI were included. Larger tumor volume was also associated with shorter DSS in both bivariate (HR 1.09, 95% CI 1.00–1.19, *p* = 0.032; Supplementary Table 2) and multivariable (HR 1.09, 95% CI 1.00–1.20, *p* = 0.043; Supplementary Table 2) analyses.

### Adverse radiation-induced events

AREs were observed in three (8%) patients, and all were peritumoral T2 signal changes on MRI 3–9 months after SRS. Two patients were asymptomatic (CTCAE grade 1); although one patient experienced increased convulsions likely related to expanding T2 signal changes and required escalation of antiepileptic therapy, his symptoms and AREs were transient and well-controlled after medical therapy alone (CTCAE grade 2).

## Discussion

Previous studies have reported on the recurrence rate of atypical meningioma following SRS, with a PFS of 33%–83% after 3 years and 20%–59% after 5 years [13–25]. However, limited studies have focused on the recurrence patterns according to intrinsic tumor characteristics, which is clinically significant as atypical meningiomas often exhibit marginal recurrences outside the radiation field [25]. Ki-67 LI provides insight into the proliferative nature of tumors, and while it has been used as a prognostic factor for postoperative recurrence in atypical meningiomas [13, 27, 28, 30, 31], few studies have evaluated Ki-67 LI as a prognosticator for atypical meningioma SRS outcomes. Shepard et al. reported that a higher LI was associated with shorter PFS after SRS in a cohort including atypical and anaplastic meningiomas classified as a Ki-67 LI of > 15% and ≤ 15% [13]. However, Kowalchuk et al. did not find an association between Ki-67 and PFS in grade 2 meningiomas [14]. Prognostic models with Ki-67 LI stratification for patterns of recurrence or DSS after SRS have also not been well-studied. Our results indicate that Ki-67 stratification was prognostic across different types of recurrence patterns (intrafield only and any form of intracranial progression) and for DSS.

For tumors with low Ki-67 LI (< 5%), no intrafield recurrences were observed, and marginal recurrences were observed only after 3 years, suggesting a more benign course similar to that of grade 1 meningiomas [12, 36]. However, tumors with intermediate and high Ki-67 LI (> 10%) were associated with intrafield recurrences with 3-year LCRs of 53% and 25%, respectively. Especially in the high LI group, patients experienced intrafield recurrence more frequently, similar to anaplastic meningioma which has 5-year PFRs of 17%–50% after SRS [5, 13, 16, 17, 20]. The prescription dose in prior studies (some including anaplastic meningiomas) has ranged from 13–15 Gy [5, 13, 16, 17, 20]; the prescription dose in the present study was 16–18 Gy. Considering that the Ki-67 LI increase was associated with a gradual increase in the risk of intrafield recurrence, it may be reasonable to gradually increase the prescription dose accordingly.

In addition to considering escalation of the prescription dose for higher Ki-67 LI, the present study’s findings suggest that treatment volumes may also need to be larger. Tumor progression tended to occur more frequently outside the 50% isodose line as demonstrated by more rapid decrease in PFR with lower rates of LCR. Marginal recurrences were frequently observed, particularly in atypical meningiomas with intermediate and high Ki-67 LI, indicating the presence of tumor cells in the dural tail or the adjacent brain parenchyma. Previous reports on SRS for meningiomas could not conclude whether the dural tail and the adjacent brain parenchyma should be included in the irradiation field [37–39]. The present study indicates that consideration for more extensive coverage including dural tail should be based on the Ki-67 LI because of the high frequency of marginal recurrence in a relatively short period after SRS for tumors with intermediate to high Ki-67 LI. Nonetheless, expansion of the radiation field should be modest.

Regarding patient survival in atypical meningioma treated with SRS, Shepard et al. found that a Ki-67 LI > 15% was a risk factor for worse survival based on univariate analysis, but there was no significant difference in multivariable analysis [13]. This was attributed to the heterogeneity of their cohort, which included anaplastic and atypical meningiomas. The present study suggests that atypical meningioma is a diverse population in terms of recurrence patterns and DSS, and that Ki-67 may be useful in predicting these outcomes. High Ki-67 LI and poor survival following treatment of atypical meningiomas is supported by the current study and prior reports as well [29, 40, 41]. In addition, the incidence of AREs after SRS was low at 8% in our cohort. However, previous reports have shown a higher occurrence of AREs with some indicating a significant risk increase after external-beam radiotherapy [13, 15]. If further escalation of radiation dose or volume coverage is considered for patients with high Ki-67 LI, this must be balanced with the potential increased risk of adverse effects in normal tissues. Recent research has shown the efficacy of multisession radiosurgery and additional hypofractionated SRS following external-beam radiotherapy [42, 43], and further research on fractionated irradiation is warranted. To this point, several prospective studies on fractionated radiotherapy for atypical meningioma and ongoing randomized trials are being conducted [44–46]. Considering that multimodal treatments should be considered in the management of atypical meningiomas, systemic therapeutic drugs have the potential to improve the prognosis of patients with atypical meningiomas [47, 48]. Additionally, several new studies on genomic alterations observed in meningiomas are providing a paradigm shift for identifying high-risk tumors [49, 50]. Prospective studies on drug therapy targeting some of these genetic mutations are also being conducted, creating hope for future genetic-based personalized medicine for atypical meningiomas [51].

## Limitations

This study had some limitations. First, this was a single-center retrospective study, and the number of patients analyzed was limited. As all patients were surgically pretreated, the heterogeneity potentially leads to selection bias. Second, the relatively short follow-up period could undermine the reliability of the results. Third, we did not perform tumor genetic mutation analysis in all cases. As a result, we cannot guarantee the exclusion of the group classified as having grade 3 anaplastic meningioma in 2021 based on *TERT* mutation or *CDKN2A/B* homozygous deletion. Therefore, future prospective studies in larger cohorts of patients are warranted to address these limitations.

## Conclusion

Ki-67 LI stratification of atypical meningioma into low (< 5%), intermediate (5%–10%), and high (> 10%) groups was correlated with outcomes after SRS in terms of LCRs, PFRs, and DSS rates. Further studies with larger sample sizes are warranted to verify these findings. Additionally, more work is needed to determine if higher doses or expanded SRS fields are needed to further decrease the risk of recurrence in patients with high Ki-67 LI.

### Supplementary Information

Below is the link to the electronic supplementary material.

Supplementary file1 (TIF 721 kb)

Supplementary file2 (DOCX 20 kb)

Supplementary file3 (DOCX 23 kb)

## Data Availability

The authors confirm that data collected for the study and analysis methods will be shared upon reasonable request from any qualified investigator.
