# Supplementary figures and images for: Ki-67 labeling index predicts tumor progression patterns and survival in patients with atypical meningiomas following stereotactic radiosurgery
Source: J Neurooncol. 2024 Feb 18;167(1):51–61. doi: 10.1007/s11060-023-04537-7 (PMC10978635; doi:10.1007/s11060-023-04537-7)

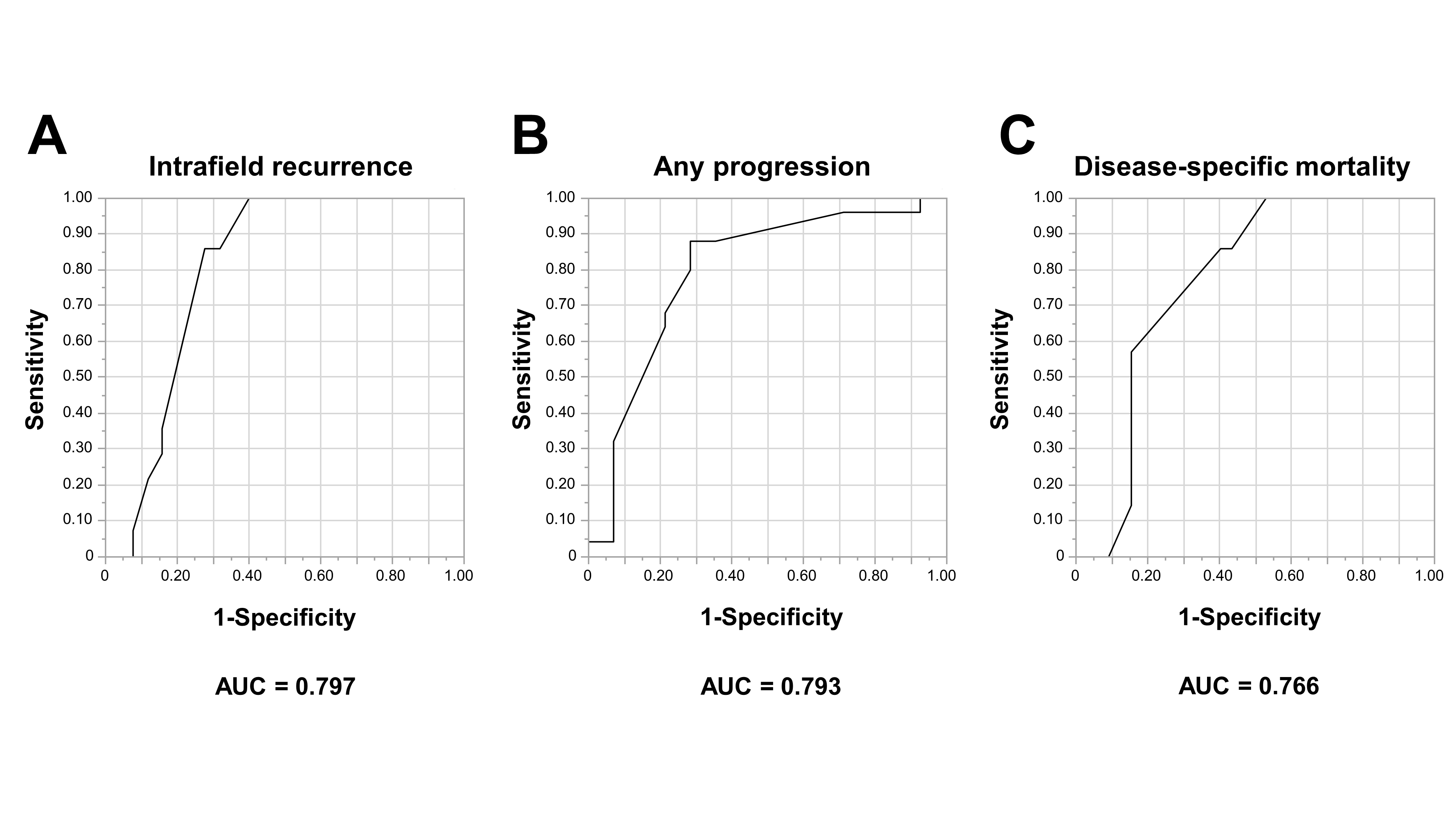

Supplement: Supplementary file 1 — Supplementary file1 (TIF 721 kb) [file 11060_2023_4537_MOESM1_ESM.tif]
