# Supplementary material for: Ki-67 labeling index predicts tumor progression patterns and survival in patients with atypical meningiomas following stereotactic radiosurgery
Source: J Neurooncol. 2024 Feb 18;167(1):51–61. doi: 10.1007/s11060-023-04537-7 (PMC10978635; doi:10.1007/s11060-023-04537-7)
Supplement: Supplementary file 2 — Supplementary file2 (DOCX 20 kb) [file 11060_2023_4537_MOESM2_ESM.docx]

**Supplementary Materials**

**Supplementary Table 1** Recurrence pattern of atypical meningiomas after stereotactic radiosurgery stratified by Ki-67 labeling index

|  | All  n = 39 | Low LI group  n = 5 | Intermediate LI group  n = 25 | High LI group  n = 9 | *p-*value |
| --- | --- | --- | --- | --- | --- |
| Intrafield recurrence | 14 (36%) | 0 (0%) | 9 (36%) | 5 (56%) | 0.116 |
| Marginal recurrence | 12 (31%) | 1 (25%) | 7 (28%) | 4 (44%) | 0.562 |
| Remote recurrence | 3 (8%) | 0 (0%) | 3 (12%) | 0 (0%) | 0.403 |

LI = labeling index.
