# Supplementary material for: Ki-67 labeling index predicts tumor progression patterns and survival in patients with atypical meningiomas following stereotactic radiosurgery
Source: J Neurooncol. 2024 Feb 18;167(1):51–61. doi: 10.1007/s11060-023-04537-7 (PMC10978635; doi:10.1007/s11060-023-04537-7)
Supplement: Supplementary file 3 — Supplementary file3 (DOCX 23 kb) [file 11060_2023_4537_MOESM3_ESM.docx]

**Supplementary Material**

**Supplementary　Table 2** Results of bivariate and multivariate analyses for risk of disease-specific survival after stereotactic radiosurgery

|  | Bivariate |  | Multivariate |  |
| --- | --- | --- | --- | --- |
|  | HR [95% CI] | *p-*value | HR [95% CI] | *p-*value |
| Age, years (continuous) | 1.03 [0.97–1.13] | 0.412 | / | / |
| Age >70 years (vs. ≤70 years) | 0.72 [0.16–3,26] | 0.668 | / | / |
| Male (vs. female) | 0.87 [0.19–4.08] | 0.863 | / | / |
| Convexity, midline (vs. skull base) | 0.82 [0.18–3.69] | 0.801 | / | / |
| Maximum diameter, mm (continuous) | 1.02 [0.97–1.07] | 0.361 | / | / |
| Maximum diameter >35 mm (vs. ≤35 mm) | 1.17 [0.26–5.27] | 0.837 | / | / |
| Volume, mL (continuous) | 1.09 [1.00–1.19] | 0.032^*^ | 1.09 [1.00–1.20] | 0.043^*^ |
| Volume >8 mL (vs. ≤8 mL) | 1.77 [0.39–7.98] | 0.457 | / | / |
| Ki-67 LI, % (continuous) | 1.04 [0.94–1.12] | 0.330 | / | / |
| High LI (vs. low LI) | NA | NA | NA | NA |
| High LI (vs. intermediate LI) | 6.27 [1.14–34.26] | 0.034^*^ | 6.55 [1.19–35.95] | 0.035^*^ |
| History of radiation therapy | 0.87 [0.17–4.55] | 0.869 | / | / |
| Salvage SRS (vs. adjuvant SRS) | 0.52 [0.10–2.68] | 0.432 | / | / |
| Central dose, Gy (continuous) | 0.98 [0.85–1.14] | 0.806 | / | / |
| Central dose >36 Gy (vs. ≤36 Gy) | 0.30 [0.04–2.51] | 0.268 | / | / |
| Marginal dose, Gy (continuous) | 0.99 [0.66–1.48] | 0.957 | / | / |
| Marginal dose >18 Gy (vs. ≤18 Gy) | 1.26 [0.28–5.71] | 0.766 | / | / |

^*^*p-*values of <0.05 are considered significant; CI = confidence interval; HR = hazard ratio; LI = labeling index; NA = not adequately calculated; SRS = stereotactic radiosurgery.
